# Higuchi Fractal Dimension of Heart Rate Variability During Percutaneous Auricular Vagus Nerve Stimulation in Healthy and Diabetic Subjects

**DOI:** 10.3389/fphys.2018.01162

**Published:** 2018-08-21

**Authors:** Ryszard S. Gomolka, Stefan Kampusch, Eugenijus Kaniusas, Florian Thürk, Jozsef C. Széles, Wlodzimierz Klonowski

**Affiliations:** ^1^Nalecz Institute of Biocybernetics and Biomedical Engineering, Polish Academy of Sciences, Warsaw, Poland; ^2^Institute of Electrodynamics, Microwave and Circuit Engineering, TU Wien, Vienna, Austria; ^3^Division of Vascular Surgery, University Clinic for Surgery, Medical University of Vienna, Vienna, Austria

**Keywords:** Higuchi fractal dimension, heart rate variability, autonomic nervous system, vagus nerve stimulation, diabetes

## Abstract

Analysis of heart rate variability (HRV) can be applied to assess the autonomic nervous system (ANS) sympathetic and parasympathetic activity. Since living systems are non-linear, evaluation of ANS activity is difficult by means of linear methods. We propose to apply the Higuchi fractal dimension (HFD) method for assessment of ANS activity. HFD measures complexity of the HRV signal. We analyzed 45 RR time series of 84 min duration each from nine healthy and five diabetic subjects with clinically confirmed long-term diabetes mellitus type II and with diabetic foot ulcer lasting more than 6 weeks. Based on HRV time series complexity analysis we have shown that HFD: (1) discriminates healthy subjects from patients with diabetes mellitus type II; (2) assesses the impact of percutaneous auricular vagus nerve stimulation (pVNS) on ANS activity in normal and diabetic conditions. Thus, HFD may be used during pVNS treatment, to provide stimulation feedback for on-line regulation of therapy in a fast and robust way.

## Introduction

Analysis of HRV represents a common tool for assessment of autonomic cardiac regulation and provides information about pathophysiological changes in various diseases (Task Force of the European Society of Cardiology the North American Society of Pacing Electrophysiology, 1996; Ashkenazy et al., 1999; Klonowski, 2007; Pierzchalski et al., 2011; Bian et al., 2012; Jiang et al., 2013; Shaffer and Ginsberg, 2017). Based on evaluation of HRV, it was recently suggested that auricular vagus nerve stimulation (VNS) positively influences the ANS by activating its parasympathetic branch (La Marca et al., 2010; Kampusch et al., 2013, 2015a,b) and deactivating its sympathetic branch (Clancy et al., 2014; Murray et al., 2016). Estimation of sympathetic and parasympathetic activity of ANS is necessary for an accurate adjustment of auricular VNS, the task that is difficult to achieve and potentially vulnerable to erroneous interpretation with standard linear methods (Skinner et al., 1992; Yeragani et al., 1993; Wagner and Persson, 1998; Klonowski, 2007; Sharma, 2009; Sassi et al., 2015).

Herein, we propose to apply a HFD algorithm (Higuchi, 1988), for assessment of ANS activity based on HRV. HFD is simple, fast and it is applicable in real-time calculations. In contrary to the linear methods, HFD can be directly applied to HRV series in time domain and it is suitable for short time series analysis, i.e., of 100–200 data points of a non-stationary signal. HFD needs to be provided with only one input parameter *k*_max_, specifying a maximal distance between the points compared in the time series. As the HFD measures the complexity of the curve that represents the analyzed signal on a plane, it always attains values between 1 and 2. The value of 1 corresponds to a regular time series (simple curve has Euclidean dimension equal 1) while for Gaussian-type noise HFD may attain different values: 1.5 for Brownian, 1.8 for pink, and 2.0 for white noise (Klonowski, 2007, 2011).

Up to date, the Higuchi algorithm was widely used in analysis of biomedical signals (Skinner et al., 1992; Klonowski, 2007, 2011; Sharma, 2009; West, 2010; Di Ieva et al., 2015; Kesić and Spasić, 2016) but only several papers presented HFD evaluation of HRV (Yeragani et al., 1993; Diosdado et al., 2010; Pierzchalski et al., 2011; Kamath, 2013; Sassi et al., 2015; Kesić and Spasić, 2016; Tavares et al., 2016; Wajnsztejn et al., 2016; Gomes et al., 2017). Hence, the aim of our research was to assess whether, based on HRV time series analysis, HFD would: (1) discriminate healthy subjects from patients with diabetes mellitus type II; (2) assess the impact of pVNS on ANS activity in normal and diabetic conditions.

## Materials and Methods

### Data

We retrospectively analyzed 56 RR time series, of 84 min duration each, from an open-label pilot study registered at ClinicalTrials.gov (no. NCT02098447). The study was approved by the local ethics committee of the Medical University of Vienna (no. 1924/2013) and by the Austrian Agency for Health and Food Safety. The RR time series were recalculated from ECG recordings obtained from nine healthy and five diabetic subjects, aged 40–80 years, with clinically confirmed long-term diabetes mellitus type II, and diabetic foot ulcer (*ulcus cruris*) lasting for more than 6 weeks (**Table 1**). Subject’s exclusion criteria were: participation in another clinical trial over the last 5 weeks before the experiment; addiction to substance abuse; autonomous nervous system dysfunction (except diabetic polyneuropathy); medical treatment with vasoactive substances; history of heart arrhythmia or presence of an active implantable device. Women in childbearing age were not included if pregnant or nursing. All diabetic subjects had a history of diabetes in average(SD) of 14(5) years. The ECGs were acquired by means of a MP36 recording system with a three-lead Einthoven II derivation (BIOPAC Systems, Inc., Goleta, CA, United States) and a sampling rate of 1 kHz, for further calculation of heart rate and HRV signals. The measurements were obtained between February 24, 2014 and April 3, 2015. The heart rate was calculated using proprietary MATLAB algorithms with manual control (normal-normal RR series, extrasystoles, and artifacts excluded manually). All subjects gave written informed consent in accordance with the Declaration of Helsinki.

**Table 1:** Demographic characteristics of healthy and diabetic subjects (*p*-values for differences in age and BMI are given) included in the study (Ref. ClinicalTrials.gov no. NCT02098447).

	Healthy subjects (*n* = 9)	Diabetic subjects (*n* = 5)
Sex (male/female)	4/5	4/1
Age (y.o.)	50.7 ± 7.2	53.8 ± 11.1 (*p* = 0.63)
BMI (kg/m^2^)	23.8 ± 3.3	34.6 ± 7.5 (*p* < 0.001)


Each of the healthy and diabetic subjects underwent four sessions of pVNS mediated via four needle electrodes, with one acting as the reference electrode, in vagally innervated regions of the right auricle (Kampusch et al., 2013, 2015a,b; Kaniusas et al., 2015). Each session consisted of five consecutive phases: B- baseline measurement (10 min), S1- first pVNS (22 min), P1- baseline measurement after the first pVNS (20 min), S2- second pVNS (22 min), P2- baseline measurement after the second pVNS (10 min). All measurements were performed at comparable daytimes.

Eleven out of 56 RR time series were excluded because of significant artifacts. The artifacts were caused by a low quality of the raw data (7 time series) or presence of cardiac arrhythmia in the signal (4 time series). Therefore, further analysis was performed on 45 RR time series (28 for healthy and 17 for diabetics).

In order to standardize the length of the series, every RR record was linearly resampled with 1 Hz. Low frequency of resampling was used to preserve the original characteristics of the signal. Higher sampling frequencies would change the shape of the original RR curve by introducing additional samples and extending the total length of the signal, subsequently affecting the estimation of real HFD values by introduction of low frequencies.

### Higuchi Fractal Dimension Algorithm

Calculations were performed by means of an in-house implementation of the HFD algorithm in MATLAB R2016b (The Mathworks Inc., Natick, MA, United States; Academic License, IBBE PAS). HRV signals were analyzed within windows of 100 data points displaced by a 50 consecutive samples across the signal, which resulted in 99 HFD values for each of the RR time series. The window of 100 data points reflected approximately 1.5 min windows in RR time series. Consecutively, every phase in each of the time series consisted of the following number of HFD values: B- 11 HFD values; S1- 25 values; P1- 24 values; S2- 25 values; P2- 14 values.

### Estimation of the Optimal *k*_max_ Parameter

**FIGURE 1:**
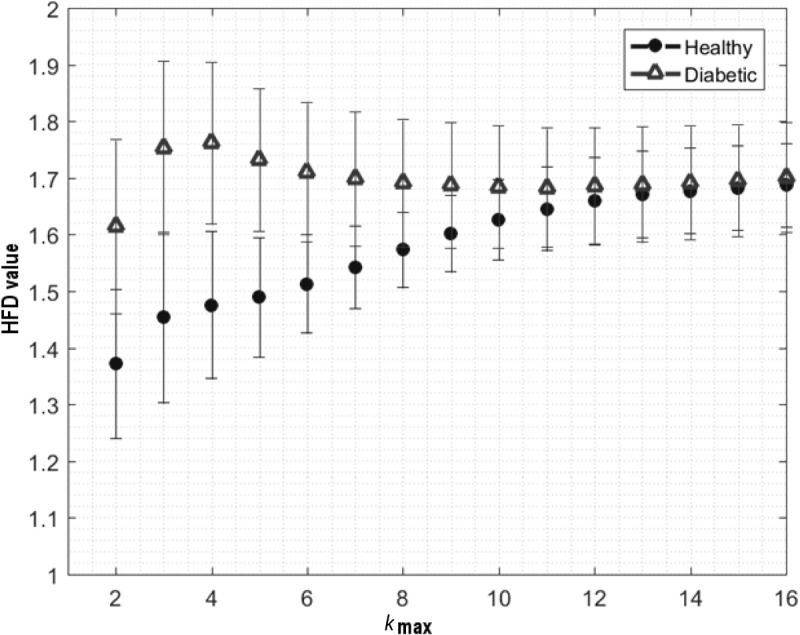
Fluctuation of aggregated average ± SD of HFD values for healthy and diabetic subjects, calculated for *k*_max_ parameter from 1 to 16. Clear differentiation between subjects obtained for *k*_max_ = 5, by means of two-sided Wilcoxon ranksum test (*p* < 7.97e–25).

In order to find an optimal *k*_max_ parameter, allowing clear differentiation between healthy and diabetic subjects, the HFD was calculated in all of the 45 RR time series, for *k*_max_ ranging from 2 to 50 (**Figure 1**). Calculation for *k*_max_ above 50 was not possible due to the window length of 100 samples – maximal distance between compared samples was less than 1/2 of the window length. Optimal *k*_max_ was chosen based on a clear separation of the mean of the aggregated HFD values for healthy and diabetic subjects, and on the minimization of the two-sided Wilcoxon ranksum test *p*-value for comparison of medians of the HFD values. Subsequent analyses were performed for HFDs calculated with the chosen parameter *k*_max_ = 5. (cf. 3.1, **Figure 1**)

### Aggregated Distributions of HFD Values – Overall

In each of the RR time series, 99 HFD values were calculated. HFDs aggregated from the time series were tested by Shapiro–Wilk normality test, to confirm non-normal distribution of the values. Afterwards, the aggregated HFD distributions were computed in healthy and diabetics from 2,772 and 1,683 HFD values, respectively. Fifth, 25th, 75th, and 95th percentiles, means, medians, SDs, skewness, and kurtosis were calculated separately in both HFD distributions, for subsequent comparison.

### Average HFD Values Across the Time Series

Mean representative vectors of HFDs were calculated in respect to time (*t*) in healthy and diabetics, for comparison by means of Wilcoxon matched-pairs signed rank test. For visual presentation of the results, the average ±95% confidence intervals of the aggregated HFD values from healthy and diabetics were plotted in function of time. Pearson’s linear correlation coefficient was calculated between the average HFDs(*t*) and the time course of the experiment in both groups. Results were considered significant if correlation exceeded 50% with a *p*-significance value <0.05.

### Average HFD Values Within the Experimental Phases

To assess whether the HFDs for healthy and diabetics are changing overtime between B, S1, P1, S2, and P2 phases, average ± SD of aggregated HFD values were calculated for each phase of the time series in each of the subjects separately. As a result, each of the subjects was represented by 5 average ± SD HFD values. Afterward, two-way analysis of variance (ANOVA) was applied to reveal significant differences between the average HFDs in healthy and diabetic groups, in respect to the phase of experiment. Bonferroni’s multiple comparison test was used in search of differences between specific phases of the time series. Whiskers-box plots of the aggregated HFDs within the phases were generated for visual representation of the results.

### Aggregated Distributions of HFD Within the Experimental Phases

To check whether pVNS changes the shape of HFD distribution overtime, 5th, 25th, 75th, and 95th percentiles, means, medians, SDs, skewness and kurtosis were calculated for the HFD aggregated distributions from B, S1, P1, S2, and P2 phases separately, for subsequent comparison. Afterwards, 10 bins-wide histograms of the aggregated HFD distributions were computed within the phases, for healthy and diabetic subjects separately. To objectively assess the magnitude of the changes caused by pVNS, contrast histograms were calculated as a ratio of difference to sum of bins heights between previously computed histograms for S1 and B; P1 and S1, S2 and P1, P2 and S2, and P2 and B phases in both groups.

### Statistical Analysis

All calculations and statistical analyses were performed by means of MATLAB R2016b.

## Results

### Estimation of the Optimal *k*_max_ Parameter

The aggregated HFD values for healthy and diabetics were found as the most statistically different for *k*_max_ = 5, by means of a two-sided Wilcoxon ranksum test (*p* < 0.001). Hence, the optimal *k*_max_ parameter, allowing clear differentiation between the healthy and diabetes group, was chosen as 5.

### Aggregated Distributions of HFD Values – Overall

Shapiro–Wilk normality test revealed that HFD values, aggregated jointly from healthy and diabetic subjects, do not form a normal distribution (*W* = 0.9745, *p* < 0.05). Characteristics of HFD aggregated distributions (i.e., 5th, 25th, 75th, and 95th percentiles, means, medians, SDs, skewness, and kurtosis) are presented in **Table 2**.

**Table 2:** Numerical characteristics of HFD aggregated distributions for experimental phases B, S1, P1, S2, P2 in healthy and diabetic subjects.

	Healthy subjects						Diabetic subjects					
	Overall	B	S1	P1	S2	P2	Overall	B	S1	P1	S2	P2
5th percentile	1.29	1.34	1.30	1.28	1.29	1.26	1.42	1.45	1.44	1.37	1.44	1.37
25th percentile	1.38	1.42	1.39	1.37	1.38	1.33	1.60	1.65	1.64	1.59	1.59	1.51
75th percentile	1.59	1.57	1.61	1.59	1.59	1.55	1.85	1.92	1.85	1.83	1.85	1.80
95th percentile	1.76	1.75	1.75	1.76	1.78	1.70	2.00	2.00	1.98	1.98	2.00	1.99
Mean	1.49	1.50	1.51	1.49	1.50	1.45	1.72	1.77	1.74	1.71	1.72	1.65
Median	1.48	1.49	1.49	1.48	1.48	1.43	1.73	1.77	1.76	1.72	1.72	1.63
SD	0.14	0.12	0.14	0.15	0.15	0.15	0.17	0.17	0.16	0.18	0.17	0.19
Skewness	0.42	0.75	0.33	0.42	0.52	0.37	–0.30	–0.64	–0.53	–0.38	–0.12	0.18
Kurtosis	2.80	3.48	2.42	2.48	2.83	3.35	2.44	2.99	2.87	2.59	2.27	2.17


### Average HFD Values Across the Time Series

**FIGURE 2:**
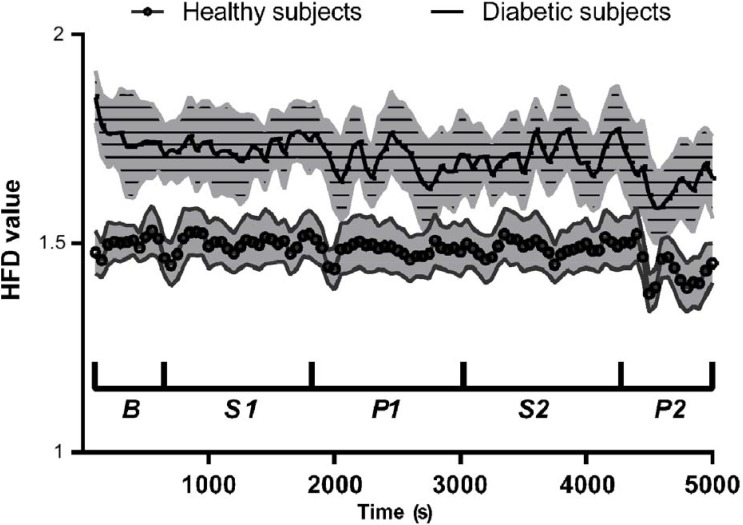
Mean ± 95% confidence intervals of HFD values during the course of experiment, for healthy and diabetic subjects separately.

Wilcoxon matched-pairs signed rank test revealed statistical differences between the average HFD(*t*) values in healthy and diabetics (*W* = 4,950, 99 pairs, *p* < 0.0001). The mean(median) difference between the groups was 0.226(0.230). Absolute Pearson’s linear correlation coefficient between the average HFD(*t*) values and the time course of experiment was larger in diabetics (*r* = –0.56, *p* < 0.0001), than in healthy (*r* = –0.44, *p* = 0.0002; **Figure 2**).

### Average HFD Values Within the Experimental Phases

**FIGURE 3:**
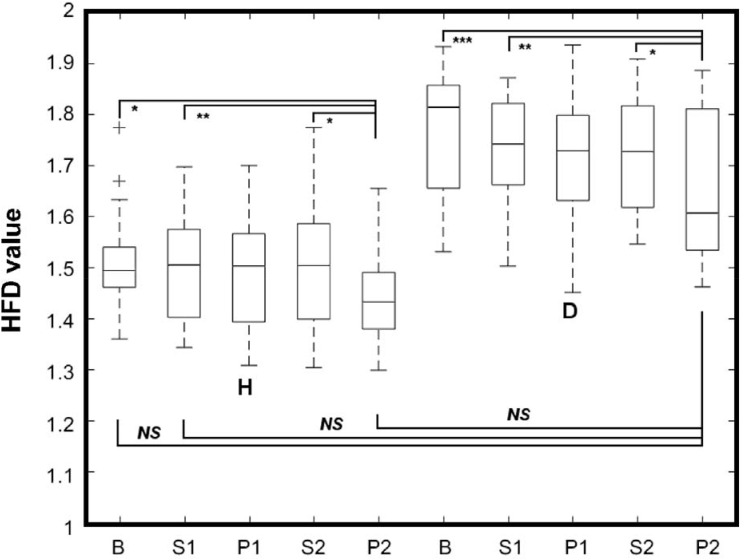
Whiskers-box plot for HFD values aggregated in respect to the phase of experiment, for healthy (H) and diabetic (D) subjects separately. Whiskers represent the range of min-max HFD values. B, S1, P1, S2, P2 – phases of the experiment. ^∗^*p* < 0.05; ^∗∗^*p* < 0.01; ^∗∗∗^*p* < 0.001.

Two-way ANOVA showed significant differences between the average HFD’s from different experimental phases, in healthy and diabetic subjects. The differences were visible between the healthy and diabetic group [*F*(1,43) = 60.79, *p* < 0.0001], and due to the experimental phases [*F*(4,172) = 10.80, *p* < 0.0001]. Bonferroni’s multiple comparisons test showed significant differences in mean HFD values between B and P2, S1 and P2, and S2 and P2 phases both in healthy (*p* < 0.05, *p* < 0.01, and *p* < 0.05, respectively) and diabetics (*p* < 0.001, *p* < 0.01, and *p* < 0.05, respectively). The mean HFD values were significantly different between healthy and diabetics in all phases (largest *p* < 0.05) except P2 in diabetic subjects, which was not different from B, S1, and P2 in healthy (**Figure 3**).

### Aggregated Distributions of HFD Within the Experiment Phases

**FIGURE 4:**
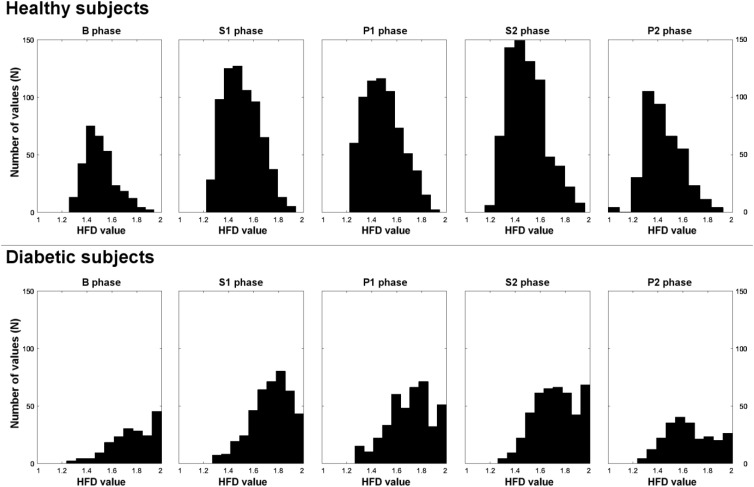
Histograms of aggregated HFD distributions from B, S1, P1, S2, P2 experimental phases, for healthy and diabetic subjects separately.

**FIGURE 5:**
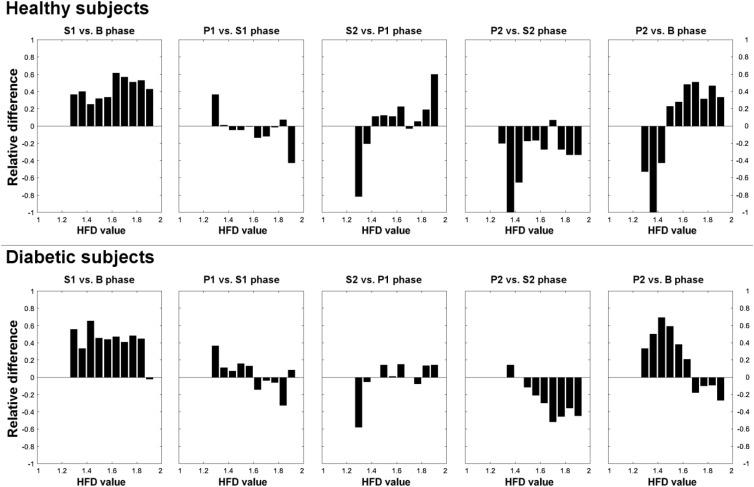
Contrast histograms of HFD distributions for comparison of S1 and B; P1 and S1, S2 and P1, P2 and S2, and P2 and B experimental phases, for healthy and diabetic subjects separately.

**Table 2** presents numerical characteristics of HFD aggregated distributions for experimental phases B, S1, P1, S2, P2 (5th, 25th, 75th, and 95th percentiles, means, medians, SDs, skewness and kurtosis) in healthy and diabetic subjects. **Figure 4** shows 10 bins-wide histograms of the aggregated HFD distributions for the phases and **Figure 5** shows contrast histograms for comparison of the distributions between S1 and B; P1 and S1, S2 and P1, P2 and S2, and P2 and B phases, for healthy and diabetics. The characteristics and the histograms were calculated in total from 4,455 HFD values. Overall mean(median) ± SD HFD values were found as 1.49(1.48) ± 0.14 and 1.72(1.73) ± 0.17 for healthy and diabetic subjects, respectively. In diabetic subjects, skewness of aggregated HFD distributions was observed to change from negative (–0.64) to positive (0.18) between the experimental phases (Pearson’s correlation coefficient *r* = 0.98; *R*^2^= 0.96, *p* < 0.01). The opposite, but not monotonic (and not significantly different from zero slope) effect was observable in the healthy group (*r* = 0.54; *R*^2^= 0.29, *p* = 0.35). Moreover, kurtosis of the distributions was found consistently dropping over the experimental phases only in diabetics.

## Discussion

This study advances knowledge in HRV analysis in healthy and diabetic subjects. By means of our implementation of Higuchi’s method, overall mean ± SD of aggregated HFD values were found higher for diabetics than for healthy subjects (**Figure 1** and **Table 2**). Significant differences in mean values of HFD aggregated distributions were found between the B, S1, and P2 phases in both groups. It is also worth to highlight that the mean HFD from P2 in diabetics was found not different from that in B, S1, and P2 phases in healthy (**Figure 3**). The results indicate no significant influence of pVNS on mean HFD during or directly after the stimulation in both groups. However, the effect seems to be delayed in time and its overall permanence was more explicit in diabetics than in healthy subjects. Moreover, skewness of the aggregated HFD distributions was rising significantly from negative to positive between the phases in diabetics, while a clear trend was not observable in healthy.

Our findings agree with the changes observed in spectral analysis of the RR time series, like the total power (TP – total variability), high frequency power (HF – parasympathetic activity), low frequency (LF – mixed sympathetic and parasympathetic activity) power, and the LF/HF ratio (sympathovagal balance) in the presented subject population (Kampusch et al., 2015b, unpublished data). TP, HF, LF, and LF/HF significantly differ between healthy and diabetic subjects at baseline and converge due to stimulation. Increases in those parameters could be shown due to stimulation in healthy and diabetics, indicating an increased parasympathetic activity and changed sympathovagal balance, like also observed in HFD analysis here. Further studies are required to understand in detail the interrelation of the evaluated HFD parameters with ANS measures. However, our findings are in accordance with previous reports regarding the HFD or detrended fluctuation analysis (Ashkenazy et al., 1999) of the HRV signal in normal conditions, congestive heart failure and heart transplanted patients (Cerutti et al., 2007), single or multiple lesions stroke (D’Addio et al., 2009), in arrhythmia (Pierzchalski et al., 2011), during meditation (Diosdado et al., 2010; Kamath, 2013), guided breathing exercises (Tavares et al., 2016), in children with ADHD hyperactivity disorder (Wajnsztejn et al., 2016), in healthy subjects immediately after physical exercises (Gomes et al., 2017), or in diabetes (Malpas and Maling, 1990).

Our results indicate a slight pVNS-induced shift of HFD values from assembled close to 2 (chaotic signal) to lower values, in diabetics. The shift was observed mostly for the HFDs above the 50th percentile of distribution and was confirmed by the change in the distribution’s skewness and kurtosis (**Table 2** and **Figure 4**). Moreover, a relative increase in HFDs below 1.6 is observable in P2, compared to B in diabetics. The effect is connected with a smaller decrease for HFDs above 1.6 (**Figure 5**). In contrary, pVNS seems to affect the whole distribution of the HFD values equally in healthy. The shape of the HFD distribution was not changed here substantially over the experiment (**Figure 4**), however, a slight increase in the higher HFDs associated with a decrease in HFDs of lower range is observable between P2 and B (**Figure 5**).

As higher HFD values correspond to the presence of higher frequencies in the signals Fourier spectrum (Yeragani et al., 1993; Klonowski et al., 2006) our observations would suggest that pVNS may increase the parasympathetic (see above) and decrease the sympathetic activity of ANS in diabetic conditions, which is in line with (Clancy et al., 2014) for healthy subjects. Such observation implies VNS-induced stabilization of ANS balance in diabetic subjects. It is worth to note that due to neuropathy a lower parasympathetic and lower sympathetic activity is generally observed in diabetes, compared to normal conditions (Task Force of the European Society of Cardiology the North American Society of Pacing Electrophysiology, 1996). Moreover, we found diabetic subjects showing larger SD of HFD values in all of the experimental phases (“more chaotic” RR signal), so pVNS seems to have a “fine-tuning” effect on ANS activity. The effect in healthy is much weaker, since pVNS seems to alter their ANS activity within the range of auto regulation capabilities.

Limitations of our study include the necessary predefinition of the input parameter *k*_max_, in advance. Herein, we have experimentally set *k*_max_ to 5. Higher *k*_max_ values would provide underestimation, while lower provide overestimation of HFD (HFD close to 1 or to 2, respectively). In both situations the distinction between the healthy and diabetic group might be not possible (**Figure 1**). Further, with respect to the included subject groups, a significant difference in BMI of healthy and diabetic subjects (**Table 1**) needs to be considered as a potential co-founding factor when analyzing ANS function. We have performed analysis of HRV time series recalculated from original ECG signals. It was previously shown that HFD may provide similar results when applied to raw data (Pierzchalski et al., 2011). Hence, it would be worth to compare HFD analysis with spectral analysis performed on the same HRV time series, or to apply both analyses to the original ECG signals from diabetic and healthy subjects. Additionally, physical activity and medication were not documented during the study for diabetic subjects.

**FIGURE 6:**
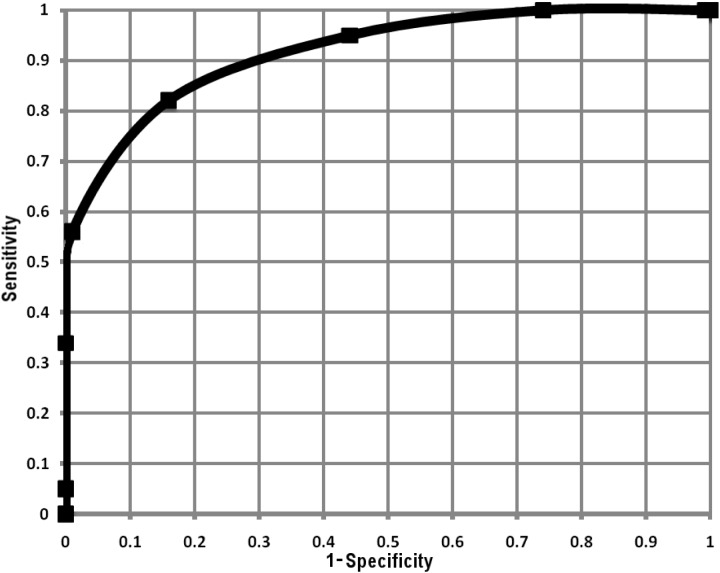
Receiver operating curve calculated from average HFD values within the experimental phases, for each healthy and diabetic subject (in total 140 HFD values for healthy and 85 for diabetic subjects).

Hence, our results indicate that HFD provides high resolution insight into ANS activity during pVNS based on HRV time series analysis. Simplicity of HFD makes the assessment of ANS activity prospectively possible in on-line systems and may bring accuracy (**Figure 6**) to both diagnostic systems and therapeutic closed-loop pVNS systems.

## Conclusion

We have shown that the HFD assesses the ANS activity and differentiates healthy from diabetic subjects, based on HRV signals. Moreover, HFD provides fast and robust distinction between action of parasympathetic and sympathetic ANS activity. Because of its simplicity, HFD may be easily used in pVNS systems to provide direct stimulation feedback for on-line regulation of therapy. Hence, our results have potential implication for patients’ care and technological advancement of pVNS therapy.

## Author Contributions

RG performed the data analysis and wrote the manuscript. SK and FT performed the data analysis and reviewed the manuscript. EK supervised the data analysis and reviewed the manuscript. JS supplied the data and reviewed the manuscript. WK designed the conception of the work, supervised the data analysis, and reviewed the manuscript.

## Conflict of Interest Statement

JS, EK, and SK own shares and receive honoraria from SzeleSTIM GmbH. The remaining authors declare that the research was conducted in the absence of any commercial or financial relationships that could be construed as a potential conflict of interest. The handling Editor declared a past co-authorship with one of the authors, WK.

## Abbreviations

ANS: autonomic nervous system
HFD: Higuchi fractal dimension
HRV: heart rate variability
pVNS: percutaneous auricular vagus nerve stimulation
SD: standard deviation
